# Nitrogen sufficiency enhances thermal tolerance in habitat-forming kelp: implications for acclimation under thermal stress

**DOI:** 10.1038/s41598-020-60104-4

**Published:** 2020-02-21

**Authors:** Pamela A. Fernández, Juan Diego Gaitán-Espitia, Pablo P. Leal, Matthias Schmid, Andrew T. Revill, Catriona L. Hurd

**Affiliations:** 1 Centro i~mar & CeBiB, Universidad de Los Lagos, Camino a Chinquihue Km 6, Puerto Montt, Casilla 557 Chile; 2 The Swire Institute of Marine Science and School of Biological Sciences, The University of Hong Kong, Pok Fu Lam Road, Hong Kong, SAR China; 3 Departamento de Repoblación y Cultivo, Instituto de Fomento Pesquero, Balmaceda 252, Puerto Montt, Casilla 665 Chile; 4 Institute for Marine and Antarctic Studies, University of Tasmania, 20 Castray Esplanade, Battery Point, Hobart, 7004 TAS Australia; 5 CSIRO Oceans and Atmosphere, GPO Box 1538, Hobart, 7001 TAS Australia

**Keywords:** Metabolism, Climate-change impacts

## Abstract

Local and global changes associated with anthropogenic activities are impacting marine and terrestrial ecosystems. Macroalgae, especially habitat-forming species like kelp, play critical roles in temperate coastal ecosystems. However, their abundance and distribution patterns have been negatively affected by warming in many regions around the globe. Along with global change, coastal ecosystems are also impacted by local drivers such as eutrophication. The interaction between global and local drivers might modulate kelp responses to environmental change. This study examines the regulatory effect of NO_3_^−^ on the thermal plasticity of the giant kelp *Macrocystis pyrifera*. To do this, thermal performance curves (TPCs) of key temperature-dependant traits–growth, photosynthesis, NO_3_^−^ assimilation and chlorophyll *a* fluorescence–were examined under nitrate replete and deplete conditions in a short-term incubation. We found that thermal plasticity was modulated by NO_3_^−^ but different thermal responses were observed among traits. Our study reveals that nitrogen, a local driver, modulates kelp responses to high seawater temperatures, ameliorating the negative impacts on physiological performance (i.e. growth and photosynthesis). However, this effect might be species-specific and vary among biogeographic regions – thus, further work is needed to determine the generality of our findings to other key temperate macroalgae that are experiencing temperatures close to their thermal tolerance due to climate change.

## Introduction

Rising levels of atmospheric CO_2_ are causing increases in air and sea surface temperatures (SSTs), with the mean SST predicted to rise by 1.4 °C to 4.8 °C by 2100^1^. With global warming, extreme high temperature events such as marine heat waves (MHWs) have also increased in frequency, intensity and duration along the World’s coastline, including the Mediterranean, Australia and Brazilian Atlantic sea^2–6^. These anomalous elevated temperatures have negatively impacted marine and terrestrial ecosystems by altering species’ composition and distribution patterns^7–9^. Such ecological changes are also severely impacting ecosystem goods and services such as fisheries, and carbon sequestration and storage^10^. The impacts of warming are considerably larger in marine systems because of their greater sensitivity to these global stressors compared to terrestrial systems^11,12^. Because of this, there is rising concern about the capacity of marine species to acclimate quickly enough to short-term variability in temperature, which will be critical for organisms to adapt and survive in a changing ocean^13,14^.

In ectothermic organisms such as plants, algae, invertebrates and lower vertebrates, temperature is the major factor regulating their physiology, growth, performance and fitness^15–20^. Therefore, changes in environmental temperatures (*T*_a_) due to climate change will trigger modifications at physiological and biochemical levels, influencing whole-organism thermal plasticity (i.e. thermal sensitivities and tolerance)^21^. These effects might be more pronounced for sessile organisms (e.g. macroalgae) than mobile ones (e.g., fish and planktonic taxa) as they are unable to escape from stressful environmental conditions, pushing them beyond their acclimation capacities^22^.

The effects *T*_a_ on biological rate processes are characterized by thermal performance curves (TPCs) (reaction norms)^16,23^. TPCs have helped to understand the effects of temperature on biological systems^24^ and to describe the thermal ecology, phenotypic plasticity and evolution of ectotherms^21,25,26^. This approach has recently been used to predict the effects of warming and short-term extreme high temperature events on physiological and ecological attributes of natural populations^24,27,28^. With warming occurring faster than predicted^29^, understanding the effects of temperature on key marine organisms such as habitat-forming species (e.g., seagrasses and macroalgae) is becoming increasingly urgent since their decline or disappearance can have substantial consequences through the entire ecosystem^8,30,31^.

Marine macroalgae (seaweeds) contribute 5–10% of global primary production and play structural and functional roles in coastal marine ecosystems, creating one of the most diverse and productive systems in the world^32–34^. Kelps (Order Laminariales) form subtidal forests in temperate and polar waters, contributing to carbon storage, macronutrient dynamics, and the biodiversity of many associated species^35^. However, a clear decline (38%) in these habitat-forming species has been observed in the past 20 years across the world^36–40^. Climate change, along with other human impacts such as overfishing and direct harvest, are considered the main reasons for this decline^39^. However, region-specific responses were also observed, suggesting that kelp’s performance is influenced by a combination of global (climate change) and local environmental drivers. Kelps are cold water-adapted species and hence vulnerable to high temperatures: As for other ectotherms, temperature exerts a large effect on their growth, survival, and reproduction^41–44^.

In addition to ongoing climate change, coastal ecosystems are threatened by local changes such as eutrophication^45,46^. The interactions between local and global drivers can be difficult to predict as these interactions can range from additive, to synergistic to antagonistic^39^. However, understanding such interactions is critical to predicting how seaweeds will fare in a future ocean. The carbon and nitrogen metabolisms in algae are tightly coupled^47–50^. Nitrogen plays important roles in regulating key enzymatic activities^51^, and is a key regulator of seaweed productivity via direct effects on cell membrane fluidity, protein production, photosynthetic machinery^49,52^, and thermal plasticity^53^. Therefore, nitrogen enrichment might ameliorate the negative effect of high temperature on seaweeds’ performance by modulating their photosynthetic and respiratory rates^5,15,53–57^. However, such positive synergistic effects can be diminished at temperatures that surpass algal thermal thresholds^5,56^, and might vary among species and populations^58^.

The giant kelp *Macrocystis pyrifera* (hereafter, *Macrocystis*) plays critical functional roles in coastal ecosystems^59^. However, in Tasmania, south eastern Australia, there has been a 90% decline in *Macrocystis* underwater forests^60^. In this region, *Macrocystis* was historically exposed to temperatures from ~12 °C to ~18 °C^61^, which agrees with the thermal window for the species across other regions^62^. However, increased warming together with progressively more oligotrophic waters (<1 µM NO_3_^−^), due to the southern shift of the East Australian current (EAC), are thought to have caused the decline in *Macrocystis*^60^. Several studies (mostly from the Pacific Northwest) have evaluated the single effects of nitrogen or temperature on *Macrocystis*’ physiology (growth, photosynthesis and nutrient uptake)^54,63–66^. However, only a few have determined the interactive effects of these two drivers^61,65,67^. To date, the regulatory effect of N on the thermal plasticity of key temperature-dependant traits under a wide range of temperatures (TPCs) is unknown for this species. Therefore, the aim of this study was to determine if N availability affects the thermal performance of physiological traits (i.e., photosynthesis, growth, nitrate uptake and assimilation) of *Macrocystis*. We hypothesized that due to the key role of N in regulating cellular and whole-organism physiological process, any potential negative effects of high temperature on *Macrocystis* performance will be ameliorated by increasing N availability. Hence the N status of the alga will play an important role in regulating thermal plasticity, i.e. N-replete blades will show greater thermal tolerance than N-deplete blades. To do this, we grew *Macrocystis* blades under low and enriched NO_3_^−^ concentrations and a range of temperatures (6–27 °C).

## Results

### Biochemical and physiological parameters of field collected samples

Biochemical measurements of field collected individuals showed an average total carbon and nitrogen percent of 24.65% and 1.27% of dry mass, respectively, with a C:N of 19.64 (electronic supplementary material, Table S1). Stable isotopes, δ^15^N and δ^13^C, were 8.60 and −14.18, respectively. NR activity was 0.43 nmol NO_3_^−^ FW g^−1^ min^−1^ (electronic supplementary material, Table S1). Other physiological and biochemical parameters such as pigment concentrations (Chl *a* and fucoxanthin (Fx)) and *F*_*v*_*/F*_*m*_ are also described in electronic supplementary material, Table S1.

### Thermal performance curves (TPCs)

**Figure 1:**
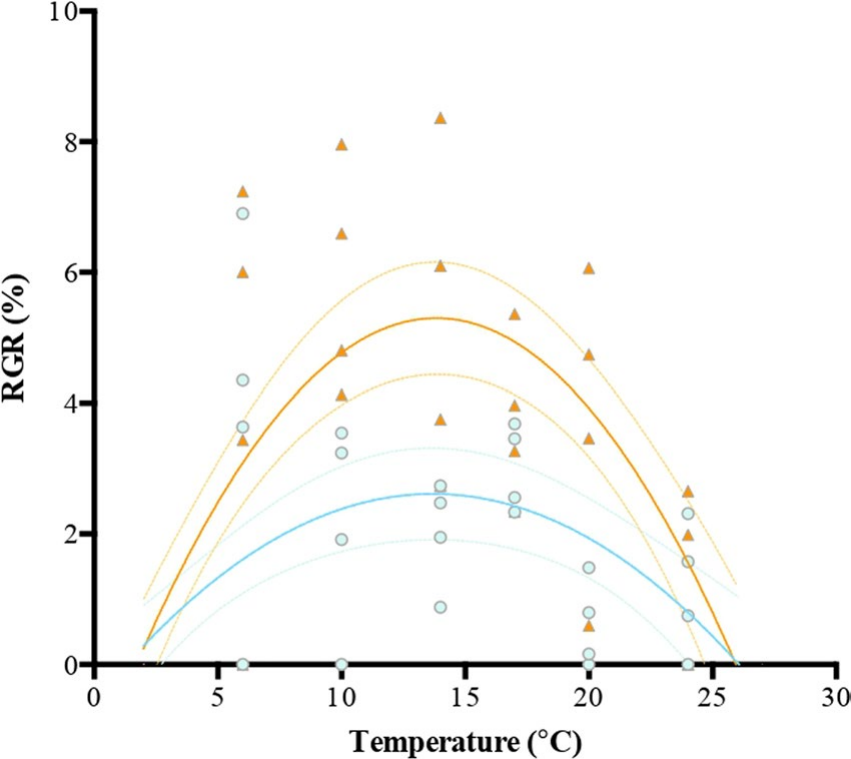
Temperature-growth response curves of *Macrocystis* blades incubated under two NO_3_^−^ concentrations (orange triangle = N-replete blades, blue dot = N-deplete blades). Each point represents one individual (n = 4 at each temperature treatment (6–24 °C)).

**Figure 2:**
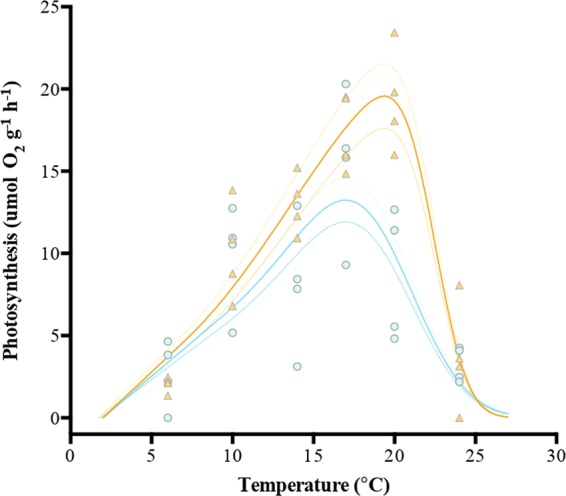
Temperature-photosynthetic response curves of *Macrocystis* blades incubated under two NO_3_^−^ concentrations (orange triangle = N-replete blades, blue dot = N-deplete blades). Each point represents one individual (n = 4 at each temperature treatment (6–24 °C)).

**Figure 3:**
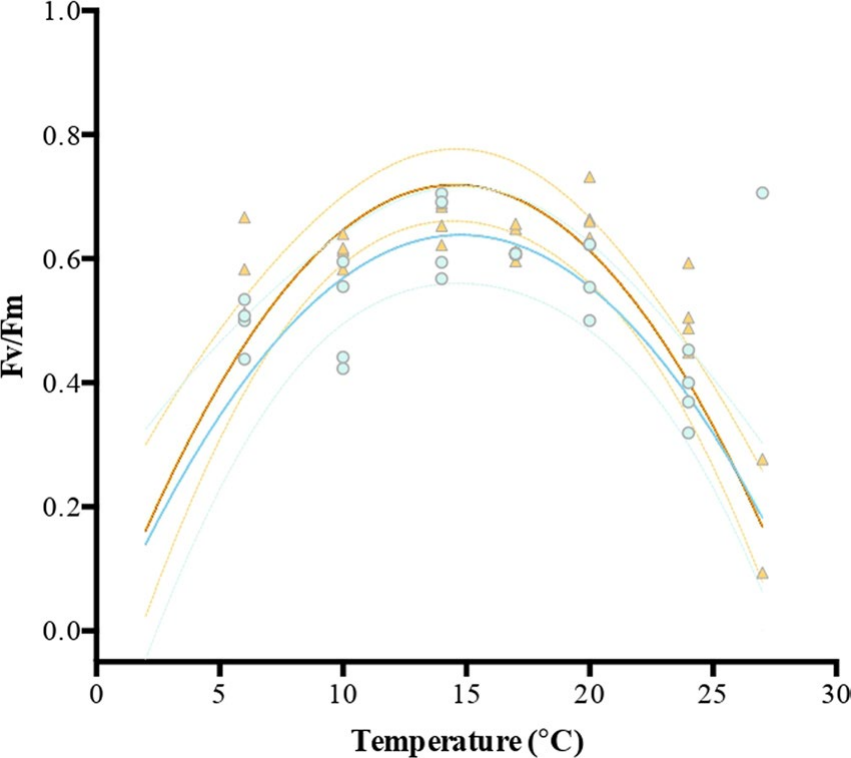
Temperature-photosynthetic efficiency (Fv/Fm) response curves of *Macrocystis* blades incubated under two NO_3_^−^ concentrations (orange triangle = N-replete blades, blue dot = N-deplete blades). Each point represents one individual (n = 4) at each temperature treatment (6–27 °C)).

**Figure 4:**
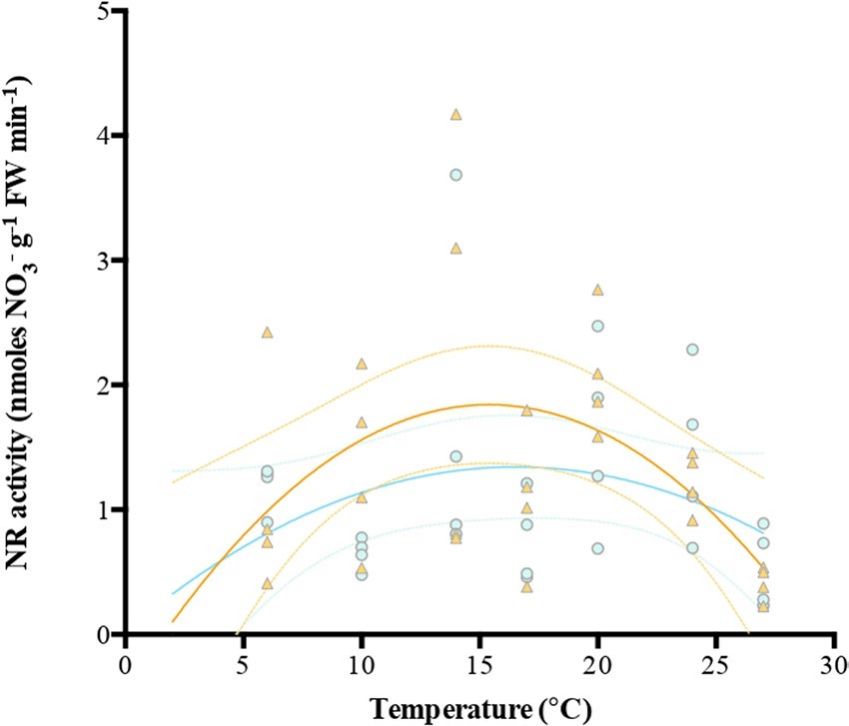
Temperature-NR response curves of *Macrocystis* blades incubated under two NO_3_^−^ concentrations (orange triangle = N-replete blades, blue dot = N-deplete blades). Each dot represents one individual (n = 4 at each temperature treatment (6–27 °C)).

Most of the physiological traits of *Macrocystis* showed the typical non-linear relationship between temperature and performance (Figs. 1–4; electronic supplementary material, Table S2). However, the effect of the internal N status on the TPCs’ shape and position varied among the physiological traits (Table 1). RGR and photosynthesis showed significant differences in the shapes and fits of TPCs between N-replete and N-deplete blades (Figs. 1 and 2) (Table 2). RGR only evidenced vertical shifts (µ_max_) whilst photosynthesis exhibited both vertical and horizontal shifts (µ_max_ and T_opt_) in the TPCs. In all of these cases, differences were explained by the considerably higher maximum rates (µ_max_) in N-replete blades than in N-deplete blades. Note that at the highest temperature (27 °C) seaweeds died in all experimental treatments and the data were not included in TPC analyses. Therefore, the TPCs are plotted for experimental treatments from 6–24 °C (Figs. 1 and 2). In contrast to the photosynthetic and growth rates, the shape and fit of *Fv/Fm* and NR activity TPCs were not significantly affected by the internal N status of the alga (Figs. 3 and 4, Table 2).

**Table 1:** TPCs traits for the physiological responses of *Macrocystis pyrifera* under different nitrate treatments (replete and deplete).

Physiological Trait	Treatment	T_opt_	CT_min_	CT_max_	µ_max_
RGR	N-replete	13.91	2.15	26.18	5.32
	N-deplete	13.86	1.86	26.72	2.57
Photosynthesis	N-replete	19.36	2.11	26.87	19.52
	N-deplete	16.86	2.13	27.13	12.91
*Fv/Fm*	N-replete	14.62	1.26	28.81	0.72
	N-deplete	14.87	1.18	28.93	0.64
NR activity	N-replete	15.38	1.78	28.93	1.84
	N-deplete	15.16	1.62	29.07	1.39

**Table 2:** Results of statistical analysis of Exact Sum of Squares F-test (non-linear models) and ANCOVA (linear models) examining the effects of the internal N status of the algae (N-replete v/s N-deplete) on the TPCs of the temperature-dependant traits: growth, photosynthesis, *Fv*/*Fm* and NR activity, and biochemical parameters of *Macrocystis pyrifera*.

Traits	Model	*F* value	*P* value
RGR	Non-linear	5.78	**0.001**
Photosynthesis	Non-linear	5.19	**0.003**
*Fv/Fm*	Non-linear	0.78	0.51
NR	Non-linear	0.94	0.42
Chl*a*	Linear	3.2	0.078
Chl*c*	Linear	2.2	0.139
Fx	Linear	1.53	0.222
N%	Linear	0.16	0.691
C%	Linear	4.08	**0.048**
C/N	Non-linear	33.9	**0.001**
δ^13^C	Linear	2.03	0.160
α	Linear	1.50	0.275
E_k_	Linear	2.18	0.191
ETR_max_	Non-linear	10.22	**0.001**

Pigment concentrations (i.e. Chl *a*, c and Fx) did not fit any of the non-linear models, suggesting a linear relationship between pigments and temperature that was not influenced by the internal N status of the alga (Table 2 and electronic supplementary material, Fig. S1).

### Total carbon and nitrogen percent, C/N ratio and Stable isotopes (δ^13^C)

Similar to pigment content, total carbon and nitrogen percent, C/N and stable isotopes followed linear a relationship with temperature (electronic supplementary material, Fig. S2). The N content and *δ*^13^*C* of *Macrocystis* blades did not differ between N-replete and N-deplete blades (similar slopes and intercepts, Tables 1 and 2), but N content showed a positive trend with increasing temperature while *δ*^13^*C* showed a negative trend. C content exhibited significant differences between experimental blades due to the negative slope in N-deplete blades and the lack of thermal influence in N-replete blades (Table 2) (slope = 0; electronic supplementary material, Fig. S2). Finally, the C/N ratio for N-replete blades showed a clear linear relationship with temperature while N-deplete blades showed a non-linear reaction curve (electronic supplementary material, Fig. S2).

### Chlorophyll a fluorescence of PSII

The initial slope (α) of the RLCs did not vary between N-replete and N-deplete blades, but decreased significantly with increasing temperature (ANOVA, P < 0.05; electronic supplementary material, Fig. S3). The E_k_ for the RLCs curves did not vary significantly between N-replete and N-deplete blades across all temperature treatments (ANOVA, P > 0.05), and ranged from 23.95 µmol m^−2^ s^−1^ at 10 °C to 76.87 µmol m^−2^ s^−1^ at 24 °C for N-replete blades, and from 32.95 µmol m^−2^ s^−1^ at 14 °C to 78.05 µmol m^−2^ s^−1^ at 24 °C for N-deplete (electronic supplementary material, Fig. S3). The ETR_max_ varied significantly among temperatures, however, neither the internal N status nor the corresponding interaction were significant (ANOVA two-ways, p = <0.001, 0.612, 0.157).

## Discussion

**Figure 5:**
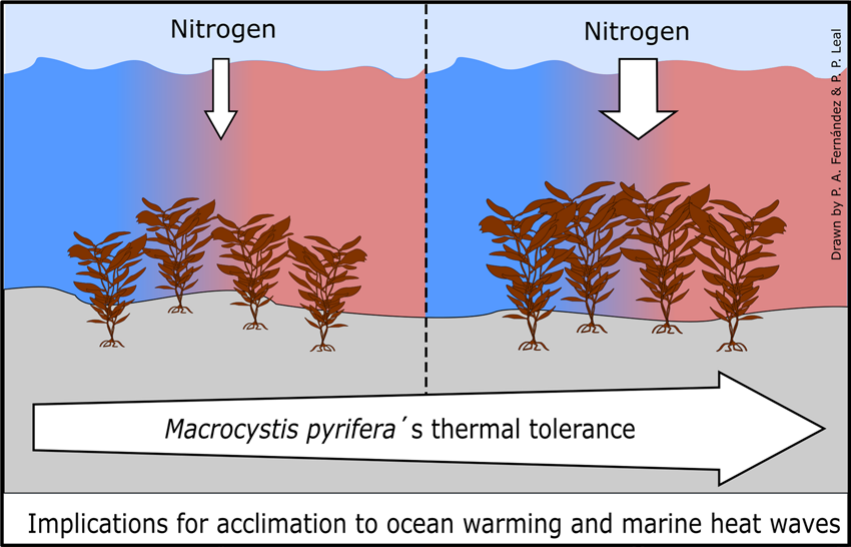
Schematic representation of the local (nitrogen) and global (warming) driver effects on the thermal plasticity of the giant kelp *Macrocystis pyrifera*. Results from the current experiments indicate that increased availability of nitrogen (wider narrow) in coastal waters can ameliorate the negative impacts of high temperature on key physiological processes (i.e. photosynthesis) in the giant kelp *Macrocystis*, increasing their thermal tolerance.

Thermal plasticity is a mechanism by which populations rapidly acclimate to warming and to extreme high temperature-related events such as MHWs^40^. Our study is the first to describe the plasticity of temperature-dependant traits of the ecologically and economically important kelp *Macrocystis*. We show that NO_3_^−^ availability and hence the internal N status of the alga modulated the thermal plasticity of *Macrocystis*, buffering the negative impact of high temperature on its physiological performance, at least over a short-term incubation (Fig. 5). This may be of great importance with short-term extreme events, such as MHWs, increasing in frequency and intensity^68^. Rapid physiological acclimation to short-term variability in temperature and/or other environmental drivers (e.g., nutrient availability) can play an important role in seaweed responses to ongoing climate change. Our results suggest that populations of *Macrocystis* which are naturally exposed to moderate inputs of NO_3_^−^ due to e.g., upwelling events or anthropogenic activities, may have a greater tolerance to high temperatures. However, populations that are exposed to limiting nutrient concentrations, like those from Tasmania, might be strongly negatively affected by OW and MHWs. We suggest that local drivers will play an important role in driving kelp responses to global change, and hence region-specific responses can be expected – as suggested by Krumhansl *et al*. (2016).

Local adaptations in thermal physiology have been recorded for macroalgal species across the latitudinal distribution of the species and ecotypes^58,69–72^ but we know little about the influence of nitrogen on the thermal performance of macroalgae. For the annual kelp *Undaria pinnatifida*, Gao *et al*.^55^ found differences in the thermal tolerance of geographically separated populations, where individuals with a higher thermal tolerance had the greatest capacity to store N. These results support our hypothesis that populations of *Macrocystis* that are naturally exposed to greater N supply will have a greater thermal tolerance than that those exposed to limiting nutrient concentrations. For microalgae, it is well documented that they are more vulnerable to high temperatures under N limited conditions compared to N-sufficient ones^73,74^. However, further studies comparing the effects of nitrogen and/or other local drivers on the thermal plasticity of populations separated geographically are urgently required to more precisely predict species’ responses to climate change.

We found that the internal N status modulated the thermal plasticity of *Macrocystis*, but its effects on TPC shapes varied among traits. Maximum photosynthetic and growth rates (µ_max_) were enhanced in N-replete blades. The positive effects of higher nitrogen availability on metabolic rates has been described in other brown kelp e.g., *Saccharina japonica*^57^. However, both the photosynthetic and growth rates of *Macrocystis* showed differences in the degree of plasticity. Only optimum temperatures (T_opt_) for photosynthesis were increased in N-replete blades while T_opt_ for growth did not change between N-replete and N-deplete blades. Also, T_opt_ for photosynthesis was higher than that for growth, which agrees with previous studies on macroalgae^41,75^. The differences observed in the thermal plasticity of growth and photosynthesis might be associated with the regulatory effect of temperature and nitrogen on each trait. For example, the photosynthetic machinery can rapidly acclimate to increases in temperature^76^, while growth is an integrated parameter that is regulated by many metabolic processes, including dark respiration, efflux of organic carbon, nitrogen uptake and assimilation^41,75^. This suggests that growth acclimation to changes in temperatures might be slower than for net photosynthesis.

Many aspects of thermal acclimation on key physiological traits are poorly studied in seaweeds compared to other marine organisms (e.g., invertebrates and corals)^77–80^ and there are currently no standardized protocols for performing thermal tolerance experiments in seaweeds. Ours is a physiological study looking into the rapid acclimation responses to temperature stress; in order to acclimate seaweeds to the experimental temperatures we used a ramping of 2 °C/hour from the acclimation temperature of 17 °C to a maximum of 27 °C and a minimum of 6 °C. We recognise that changes of 10 °C in a span of 5 h are unlikely to occur in the shallow subtidal system from which we collected *Macrocystis*, and although this may have contributed to increased physiological stress, the experiments were designed to develop the thermal performance curves (TPCs) of key physiological traits. Other studies have performed thermal ramps of 5 °C per day^81^, 3 °C per three days^82^ and some have not performed any thermal acclimation before the start of experiments^56^. In order to make more realistic long-term predictions to climate change (i.e. OW and MHWs), development of appropriate protocols for thermal stress experiments (i.e. thermal ramping and acclimation procedure) are required, understanding that these conditions might vary among regions, depending on daily and seasonally local variability (i.e. in temperature).

Changes in thermal tolerance involve various adjustments in algal metabolism, in which nitrogen plays a critical regulating role^83^. These adjustments include increased production of heat shock proteins (HSPs), which are important to tolerate temperature-stress conditions^84–86^ and changes in fatty acid composition and lipids of the thylakoid membrane^41,87^. We did not measure HSPs across the experimental treatments, but further investigation of the fatty acid and lipid composition in selected experimental treatments (6, 17 and 24 °C) (M. Schmid unpublished data) showed that under high nitrate concentrations, *Macrocystis* maintains a high proportion of polar lipids (PL) and shows no increase in free fatty acids (FFAs). Under low nitrate concentrations, however, PL decreased markedly with increasing temperatures, with a concomitant increase in FFAs. PL are key component of cellular membranes^88^, and hence a decrease in their proportion can negatively affect membrane stability at high temperatures. These results showed that nitrogen can rapidly influence lipid metabolism, and is a likely mechanisms by which *Macrocystis* acclimates to short-term thermal stress.

The thermal plasticity of *Fv/Fm*, which is often used as an indicator of photosynthetic stress and photoinhibition^89^, was not influenced by the internal N status of the algae, suggesting that the initial stage of the light reaction of photosynthesis (i.e. the transport of electrons through PSII) was stable under both low and enriched NO_3_^−^ concentrations. In phytoplankton^90,91^ and the green macroalga *Ulva sp*.^92,93^, N limitation negatively affects PSII efficiency, indicated by a decline in *Fv/Fm*, likely due to decreases in protein synthesis and reaction centres^90^. However, we observed no effect of the internal N status of the alga on PSII efficiency (i.e., *Fv/Fm*, ETR_max_, E_k_ and α) and nor on the photosynthetic pigments, at least over the short term incubation. Our results, along with those of Mabin *et al*.^61^ who found no effect of NO_3_^−^ (0.5–3.0 µM) on the PSII parameters *Fv/Fm* and rETR_max_ of *Macrocystis*, suggest that seawater NO_3_^−^ concentrations were above those required for the efficient functioning of PSII. In south-eastern Australia, this species is naturally exposed to low NO_3_^−^ concentrations and hence the functioning of the PSII might be locally adapted to low nutrient concentrations.

Availability of NO_3_^−^ can directly affect NR activity by regulating the synthesis and degradation of the protein^94^, and it can be strongly regulated by internal NO_3_^−^ pools^95^. In macro- and microalgae, NR activity is also responsive to changes in temperature^96^, with thermal plasticity (T_opt_) varying between phytoplankton species^97–99^. However, there are no studies describing the regulating role of NO_3_^−^ on NR thermal plasticity in macroalgae or phytoplankton species. The NR thermal plasticity of *Macrocystis* was not influenced by the internal N status of the algae. T_opt_ (15 °C) for NR in N-replete and N-deplete blades was similar to those described for phytoplankton species, ranging between 10 to 20 °C, which are typically close to the T_opt_ for growth^97^. This agrees with our study where T_opt_ for NR was similar to that for growth (13–14 °C). The lack of effect of NO_3_^−^ in regulating the NR thermal plasticity of *Macrocystis* may be due to the high intraspecific variability observed among individuals (activities ranged from 0.77–4.0 nmol NO_3_^−^ g^−1^ FW min^−1^), or that NR activity was not limited by the NO_3_^−^ concentration in the treatments. Similar to higher plants, two NR forms, one inducible and one constitutive, may occur in macroalgae^100^. It is possible that the inducible NR might be regulated by external environmental parameters, and the constitutive form maintains a constant activity rate^101^, which could explain our results. Thus, even when external NO_3_^−^ concentrations are low, NR activity remains active. However, the regulation of the constitutive NR form has not been studied in macroalgae. Although we did not observe an increase in NR activity in N-replete blades, higher growth rates were observed across most of the temperature treatments, suggesting that more NO_3_^−^ was assimilated and converted to N to support growth. Moreover, *Macrocystis* from New Zealand can rapidly respond to changes in N availability, up-regulating its N metabolism^95^. However, similar to PSII, NR activity for *Macrocystis* from Tasmania might be adapted to the local low ambient nutrient concentrations, and thus responses to environmental variability (physiological plasticity) might be distinct from other populations across the world.

Our results showed that thermal plasticity, tolerances and sensitivities, can vary markedly among physiological traits, with some traits responding faster than others to short-term variability in environmental temperature. Similarly, Wernberg *et al*.^69^ showed that in three habitat-forming seaweeds (*Sargassum fallax, Ecklonia radiata, Scytothalia dorycarpa*) optimum temperature for photosynthesis ranged from 23 °C–25 °C, depending on the species; however, no optimum temperatures were detected for respiration. Previous studies on ectothermic animals have shown similar variability among traits (e.g., respiration, growth)^27^. These results highlight the importance of selecting the right traits for predicting the effects of warming on the whole organism^27^. For macroalgae, photosynthesis can rapidly respond to changes in temperature, which agrees with our study, and it is considered a good proxy to compare thermal performance between species^75^. However, for long term predictions, photosynthesis might not be the best parameter to use because it over estimates the upper thermal tolerances for long term growth^75^. Studies describing thermal plasticity of different traits in macroalgae are urgently needed to identify the most relevant traits to precisely predict and compare the effects of ongoing warming at the organism and population level.

Recent studies have highlighted the importance of plasticity and local adaptation in macroalgal responses to warming, suggesting that some ecotypes might be more resilient or vulnerable to high temperatures than others, depending on the conditions to which they are usually exposed^40,86,102^. Previous studies have illustrated the physiological plasticity of *Macrocystis* across its wide geographical distribution. For example, marked differences in thermal tolerance and N storage capacities have been observed in populations that are geographically isolated^66,103–105^. Similarly, distinct thermal tolerances and survival abilities have been observed in microscopic life stages from populations locally exposed to a different gradient of temperature (warmer vs. cool temperate sites)^106^. These results suggest that populations that are naturally exposed to highly variable thermal and nutrient regimes can have different responses to future oceanic conditions compared to populations that are exposed to more stable environmental conditions. Further studies linking molecular (e.g., expression of heat-shock-proteins) with physiological responses will provide a better understanding of macroalgal plastic and adaptive capacities to respond to climate change.

## Materials and Methods

### Seaweed collection

Sampling was performed at Bruny Island (45°47′S, 170°43′E), Tasmania, Australia, in March 2016. At the time of collection, temperature and NO_3_^−^ concentrations in surface waters ranged from 18.7–19.9 °C and from 0.14–0.53 µM NO_3_^−^, respectively. Also, temperatures were 3–4 °C above the average of previous summers due to the most intense and longest MHW recorded in the Tasman Sea^107^. A total of 80 young blades (the 2^nd^ and 3^rd^ blades below apical scimitar) were collected from different individuals of *Macrocystis* (3–4 blades from each of 26 sporophytes). Blades were transported to the laboratory in an insulated container with ambient seawater. At the laboratory, blades were gently rinsed and cleaned with 0.5 µm filtered natural seawater (NSW) of any visible epibionts by gently brushing. Each of the 80 blades were cut to a similar size of 11 cm × 3.5 cm (fresh initial weight 1.0 ± 0.2 g), at 2 cm from the neumatocyst/blade junction (meristematic zone). Initial physiological measurements on field collected samples (electronic supplementary material, Table S1) showed that seaweeds were healthy at the start of the experiment. Then, blade sections were incubated for 12 h to allow marginal wounds to heal, in transparent 2 L-jars (0.5 µm filtered NSW at 17 °C). Mixing in the culture tanks was provided by pumping air. Eight blade sections were used to assess their initial physiological status (i.e. photosynthetic parameters, growth, nitrate reductase (NR) activity and nitrogen and carbon content) as described below.

### Pre-experimental incubations under low and enriched-NO_3_^−^ concentrations

After the 12 h healing time, 72 blade sections were incubated for a further 3 days under low (5 µM NO_3_^−^; n = 36) and enriched-NO_3_^−^ concentrations (80 µM NO_3_^−^; n = 36) to obtain *Macrocystis* blades with different nitrogen status, i.e. deplete and replete, respectively^95^. Six blade sections were placed into each of twelve 2 L-culture tanks, six containing low-NO_3_^−^ SW and the other six containing enriched-NO_3_^−^ SW. A 20 mM NaNO_3_ solution provided the desired NO_3_^−^ concentrations in each culture tank, and 100 mM PO_4_^−^ was used to avoid P limitation through the experiment (5:1 N:P). Seawater mixing was provided by pumping air. A saturating photon flux density of 120–130 µmol m^−2^s^−1^ was provided overhead by florescent white tubes (Envirolux CE F28T5/4100K-120477 240V) set on a 12L:12D photoperiod. Incident light was measured using a Li-Cor LI-1400 data logger equipped with a flat underwater radiation sensor LI-192. SW samples (10 mL) were taken every day before and after renewing the medium to monitor NO_3_^−^ concentrations (electronic supplementary material, Table S1). Prior to the experiments and during the pre-experimental conditions, the cultures were maintained in a temperature-controlled room at 17 °C.

### Temperature and nitrate incubations, and experimental design

After the pre-experimental incubation, N-deplete (blades coming from the 5 µM NO_3_^−^ treatment) and N-replete blades (blades coming from the 80 µM NO_3_^−^ treatment) were haphazardly selected and placed into each of 64 Erlenmeyer 250 mL flasks, containing either low (n = 32) or enriched-NO_3_^−^ SW (n = 32). After that, each 250 mL culture flask was randomly assigned to one of the seven temperatures treatments, 6–10–14–17–20–24–27 °C, with four replicates for each experimental treatment. Blades were gradually acclimated from the pre-incubation temperature (17 °C) to the experimental temperatures. Thermal ramps were performed with linear temperatures changes of 2 °C per hour over a span of 5 h. Although the temperature range, 6 °C to 27 °C, and the thermal ramp does not fully coincide with the conditions experienced by the species in the field, the temperature range and ramping were selected to estimate the short-term thermal acclimation and precisely develop the TPCs for the specie. The culture flasks under each temperature treatment were maintained in a controlled temperature water bath for three days and subjected to a 12L:12D photoperiod under a saturating light intensity of 120–130 µmol m^−2^s^−1^ provided and measured as described in the pre-experimental incubations. SW was changed daily and mixed by pumping air. Temperature and light conditions within each temperature treatment were monitored continuously using HOBO pendant temperature/light data loggers (64K-UA-002-64). SW samples (10 mL) were taken every day before and after renewing the medium to monitor NO_3_^−^ concentrations in each treatment (electronic supplementary material, Table S1). After the 3-day incubation, *Macrocystis* blades were harvested to determine their physiological and biochemical responses (i.e. photosynthesis, growth, nitrate assimilation, and carbon and nitrogen content).

### Physiological and biochemical parameters

Photosynthetic rates expressed as oxygen evolution were measured on the last day of the temperature/nitrate experiment, for each of the 56 experimental *Macrocystis* blades (n = 4 for each treatment combination). To do this, each blade was incubated separately in a biochemical oxygen demand (BOD) bottle, containing 266 mL of filtered 0.5 µm SW. A control BOD bottle without seaweed was also carried out. BOD bottles were placed on the top of an orbit shaker table set at 100 rpm to provide water movement. Dissolved oxygen (DO) was measured at the start and after 1 h of incubation using an optical dissolved oxygen sensor (Hach LBOD10101) and a DO meter (HQ40d Hach). Photosynthetic rates were determined under a saturating light of 120–130 µmol m^−2^s^−1^ that was provided overhead by florescent white tubes (Envirolux CE F28T5/4100K-120477 240 V). For each temperature treatment, BOD bottles were put inside a transparent plastic box, where the temperature was controlled by an aquarium heater set up at each temperature. Photosynthetic rates were estimated from the initial and final oxygen concentrations (mg/l), and standardized by the fresh weight (g) of each blade and the incubation volume (l).

After measuring photosynthetic rates, chlorophyll *a* fluorescence of photosystem II was measured using a Pulse Amplitude Modulation fluorometer (diving-PAM, Walz, Germany). *Macrocystis* blades from the different treatment combinations were dark-adapted for 20 min before exposure to the PAM’s photosynthetic active radiation (PAR, 0–422 μmol photons m^−2^ s^−1^). Rapid light curves (RLCs), relative ETR (rETR) versus irradiance, were conducted right after dark adaptation. Calculations of rETR of algal samples were estimated using the equation:

$$rETR=Y(II)\times {\rm{EPAR}}\times {\rm{A}}\times 0.5,$$

where Y(II) is the quantum yield of photochemical quenching, E the incident irradiance of PAR, A the average ratio of light absorbed by algal tissue (0.8 for kelps) and the factor 0.5 assumes that 50% of the all absorbed energy has been utilized by PSII^108^. The rETR-RLCs were fitted according to Eilers and Peeters (1988), so that the light saturation (E_k_), initial slope (α) and the maximum ETR (ETR_max_) parameters were calculated from each curve. The optimum quantum yield (*Fv/Fm*), which represents a good indicator of maximal algal photosynthetic efficiency^109^, was calculated right after the dark adaptation.

Relative growth rates (RGR, % days^−1^) were estimated after measuring photosynthetic rates and Chlorophyll *a* fluorescence by the difference in fresh weight (FW) after 3 days of incubation, using the formula:

$$RGR={\rm{In}}(\frac{Wt}{{W}0})\times t-1\times 100,$$

where W_0_ is the initial FW and W_t_ is the final FW after *t* days of incubation. The FW was estimated after blotting the blade section gently with tissue.

After the growth measurement, blade sections, from each experimental treatment, were cut along the blade into four pieces in order to assess NR activity, C and N content and pigment concentrations. Tissue samples for NR activity (0.21–0.30 g FW), pigment analysis (0.10–0.15 g FW) were immediately frozen in liquid N_2_ and stored at −80 °C until further analyses. Tissue samples for C and N content and stable isotopes (0.008–0.010 g FW) were oven dried for 48 h at 60 °C.

NR activity was measured by nitrite production in an *in vitro* assay^110^. NR extraction methodology is described in detail in Fernandez *et al*.^95^. Briefly, NR was extracted in a 200 Mm Na-phosphate buffer (pH 7.9), containing 3% w/v BSA, 0.3% w/w polyvinylpyrrolidone (PVP), 2 Mm Na-EDTA and 1% w/v Triton X-100 (all Sigma, St Louis, MO, USA). The content of photosynthetic pigments (chlorophyll *a* and fucoxanthin) was analysed using methods described in Seely *et al*.^111^, Wheeler^112^, and Stephens and Hepburn^113^, where dimethyl-sulfoxide (DMSO) was used for the primary extraction and acetone for the secondary. Tissue samples for C and N content and stable isotopes from field collection and pre-experimental incubations, and temperature/nitrate experiments were determined according to Cornwall *et al*.^114^.

### Seawater analyses

Nitrate concentrations were analyzed using a QuickChem 8500 series 2 Automated Ion Analyzer (Lachat Instrument, Loveland, CO).

### Data analysis

Prior to analyses, we tested for normality and homoscedasticity for all variables, using the Lilliefors and Levene tests, respectively. Data was transformed either by log_10_ or by square root to fulfil the requirements for parametric tests. Comparisons of parameters measured as part of the chlorophyll a fluorescence of PSII, were done via analysis of variance (ANOVA). When differences in the means were significant at the P < 0.05 level, they were also tested with a posteriori Tukey’s test (HSD). Statistical analyses were performed with R 3.0.2 software and the package lme4^115^ and in the GraphPad Prism software (v.7.03).

**Figure 6:**
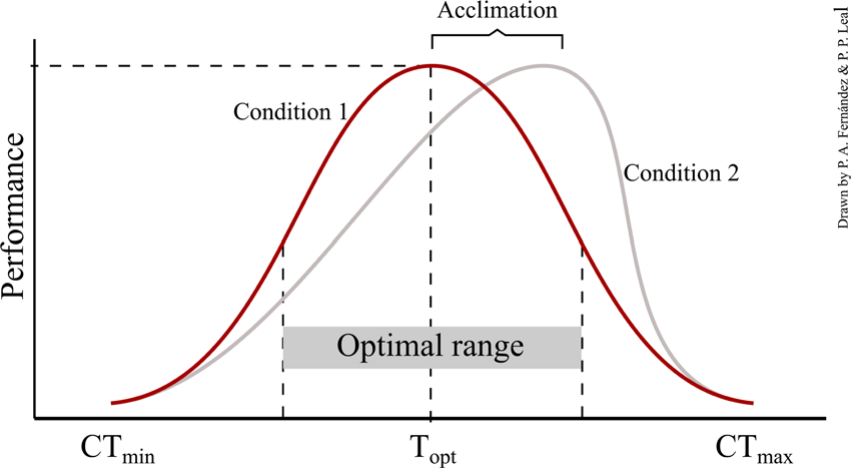
Typical TPCs describing the critical thermal minimum (CT_min_) and critical thermal maximum (CT_max_), at which physiological responses (e.g., photosynthesis, respiration) are possible. The thermal optimum (T_opt_) is the temperature at which the physiological response reaches its maximum performance, and the optimal range represents the temperature range at half the maximum of performance (based on Angilletta *et al*. 2002, Sinclair *et al*. 2016). Both curves are representing plasticity in response to some environmental drivers (i.e. temperature), and the shift between curves (red to grey) represent a plastic adjustment to a different condition (e.g., nitrogen).

The effect of temperature on physiology and performance was described by a continuous nonlinear reaction norm (i.e. TPC) (Huey *et al*. 1999). Variation in the parameters of the TPCs (i.e. the optimal temperature - T_opt_; the thermal breadth - T_br_; the maximal performance - µ_max_; and the upper and lower limits of temperature at which traits expression decrease - CT_min_ and CT_max_) was used to describe mechanistically the variation of thermal sensitivities and tolerances of natural populations (see Gaitan-Espitia *et al*. 2013, 2014) (Fig. 6). Here, we used the TableCurve2D curve-fitting software (version 5.01; Systat Software, Inc.) and the GraphPad Prism software (v.7.03) for model fitting. TPC parameters (µ_max_, T_opt_, CT_min_ and CT_max_) were extracted from the best models (see below for details). The physiological characteristics of critical thermal maximum (CT_max_) and minimum (CT_min_) were derived numerically as the intersection points of the resulting thermal performance curve with the temperature axis (μ = 0).

The fit of several linear and non-linear functions (e.g., Gaussian, Quadratic Lorentzian, Weibull) that could describe organismal performance as a function of temperature was analyzed using the Akaike Information Criterion (AIC) (Angilletta 2006). The AIC represents a balance between the likelihood explained by the model and the number of model parameters, with the best model minimizing AIC^116^. Thermal-dependent traits obtained from the TPCs were analyzed using a linear modelling approach. The effects of temperature and NO_3_^−^ (fixed effects) were evaluated through confidence intervals (CI) computed from the likelihood profile^115^. In addition to CI, parameters and shapes of the TPCs were analyzed and compared using AIC and the Extra Sum-of-Squares F test. For linear models, the slopes and intercepts were compared using an analysis of covariance (ANCOVA) F-test.

## Supplementary information

Supplementary information.
