# Supplementary material for: Nitrogen sufficiency enhances thermal tolerance in habitat-forming kelp: implications for acclimation under thermal stress
Source: Sci Rep. 2020 Feb 21;10:3186. doi: 10.1038/s41598-020-60104-4 (PMC7035356; doi:10.1038/s41598-020-60104-4)
Supplement: Supplementary file 1 — Supplementary information. [file 41598_2020_60104_MOESM1_ESM.pdf]

Supporting information

Nitrogen sufficiency enhances thermal tolerance in habitat-forming kelp: implications for acclimation under thermal stress

Pamela A. Fernández, Juan Diego Gaitán-Espitia, Pablo P. Leal, Matthias Schmid, Andrew Revill, Catriona L. Hurd

This PDF file includes:

- Table S1 to S2
- Figs. S1 to S3

Table S1: Table summarizing the biochemical and physiological parameters from field collected samples, and after the pre-experimental incubations (N-replete and N-deplete blades), and nitrate concentrations. The values are average (n=5) ± AV.

**Parameters**	**Field collected samples**	**Pre-experimental incubations**	
		**NO_3^--low SW**	**NO_3^--enriched SW**
$\delta^{15}N$	8.60 ± 0.33	6.41 ± 2.5	7.46 ± 2.49
$\delta^{13}C$	-14.18 ± 1.23	-13.50 ± 0.80	-12.89 ± 1.39
N (%)	1.27 ± 0.17	1.39 ± 0.40	1.58 ± 0.169
C (%)	24.65 ± 1.72	23.80 ± 3.00	24.37 ± 1.55
C/N	19.64 ± 2.89	17.82 ± 4.51	15.43 ± 0.66
Growth rate (% day^{-1})	-	1.28 ± 0.52	2.13 ± 0.86
NR activity (nmol NO_3^- FW g^{-1} min^{-1})	0.43 ± 0.24	1.05 ± 0.15	2.65 ± 1.17
Photosynthetic rate (O_2 mg L^{-1} FW g^{-1} h^{-1})	-	0.45 ± 0.05	0.55 ± 0.01
Fv/Fm	0.71 ± 0.01	0.59 ± 0.01	0.69 ± 0.004
Chl *a* (mg g FW)	0.41 ± 0.02	0.45 ± 0.03	0.38 ± 0.04
Fx (mg g FW)	0.22 ± 0.005	0.24 ± 0.01	0.20 ± 0.02
SW [NO_3^-] (µM) initial	0,28±0,16	5,51 ± 0,43	76,39 ± 15,5
SW [NO_3^-] (µM) after 24h		0,98 ± 0,23	0,92 ± 0,26
**Temperatures/N experiments:**			
SW [NO_3^-] after 24h	6°C	1,94±2,01	0,97±0,19
	10°C	0,91±0,40	1,86±1,92
	14°C	1,14±0,17	3,08±2,53
	17°C	1,08±0,54	2,67±3,25
	20°C	1,26±0,35	1,82±0,97
	24°C	3,21±1,64	29,00±18,65
	27°C	9,03±2,56	65,78±22,03

Table S2. Comparison of functions used to describe the thermal performance curves for traits of *Macrocystis pyrifera* under two NO_3^- treatments.

Trait	Treatment	Function	K	r^2	Adj r^2	Absolute SS	Sy.x	AICc	λ_i
RGR	N-replete	**Quadratic**	4	0.6847	0.6694	93.27	1.605	42.08	0
		Gaussian	4	0.6503	0.6329	103.5	1.589	46.68	4.6
		Lorentzian	4	0.5781	0.5576	124.8	1.745	54.9	12.82
	N-deplete	**Quadratic**	4	0.4264	0.3984	58.8	1.198	21.78	0
		Lorentzian	4	0.321	0.2878	69.6	1.303	29.21	7.43
		Gaussian	4	0.3851	0.3551	63.03	1.24	46.68	24.9
Photosynthesis	N-replete	**GMG**	5	0.9065	0.879305	294.2	2.72	33.17786985	0
		EMG	5	0.8921	0.865337	303.7	2.94	34.92264283	1.744772981
		Quadratic	4	0.8841	0.856442	309.5	3.14	35.2342802	2.6341042
		Weibull	5	0.8734	0.847198	322.8	3.38	36.96794571	3.790075863
		Gaussian	4	0.814	0.78958	345.4	3.45	38.55172491	5.373855059
		Lorentzian	4	0.8059	0.781723	381.3	3.83	39.07217117	5.894301325
	N-deplete	**GMG**	4	0.7428	0.71281	309.5	2.982	39.34883653	0
		Gaussian	4	0.6339	0.595866	346.2	3.649	39.86867021	0.51983368
		Quadratic	4	0.6452	0.606488	348.1	3.662	39.929757	0.58092047
		Lorentzian	4	0.632	0.59408	376.5	3.805	39.98400062	0.635164087
		Weibull	5	0.537	0.50478	387.3	3.953	42.47279818	3.123961644
Fv/Fm	N-replete	**Quadratic**	4	0.7917	0.7757	0.2825	0.1042	-124.6	0
		Gaussian	4	0.6833	0.659	0.4294	0.1285	-112.5	12.1
		Lorentzian	4	0.6037	0.5732	0.5374	0.1438	-106	18.6
	N-deplete	**Quadratic**	4	0.6003	0.5696	0.5075	0.1397	-107.7	0
		Gaussian	4	0.558	0.524	0.5614	0.1469	-104.7	3
NR activity	N-replete	**Quadratic**	4	0.2811	0.2258	18.4	0.8412	-3.532	0
		Gaussian	4	0.2557	0.1985	19.05	0.8559	-2.524	1.008

	Lorentzian	4	0.2318	0.1727	19.66	0.8695	-1.608	1.924
N-deplete	**Quadratic**	4	0.2487	0.1986	15.69	0.7232	-15.1	0
	Gaussian	4	0.2118	0.1592	16.46	0.7408	-13.52	1.58
	Lorentzian	4	0.1879	0.1338	16.96	0.7519	-12.53	2.57

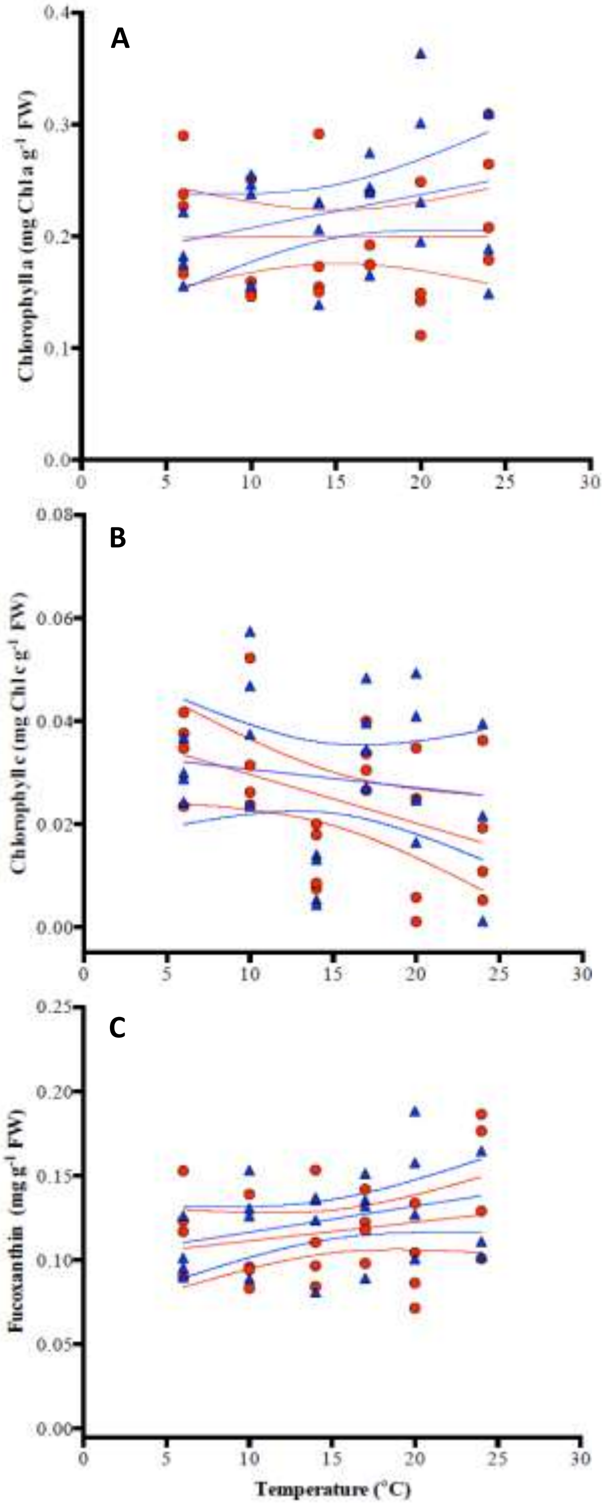

Fig. S1. Photosynthetic pigments (A) Chla, (B) Chlc, (C) Fucoxanthin of *Macrocystis* blades incubated under two NO_3^- concentrations (red dot = N-deplete blades, blue triangle = N-replete blades). Each point represents one individual (n = 4 at each temperature treatment (6-27°C)).

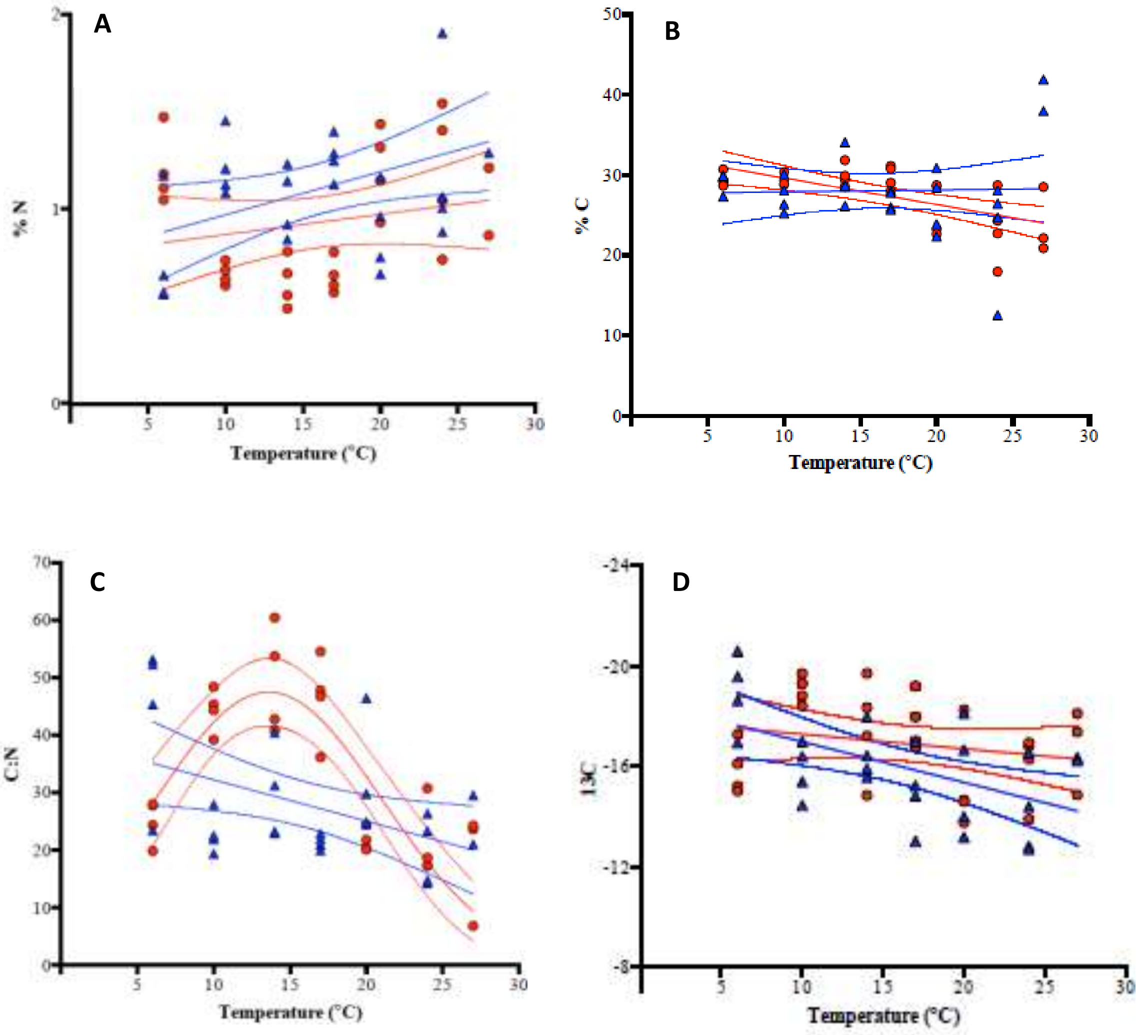

Fig. S2. Biochemical parameters (A) N content, (B) C content, (C) C/N, (D) δ^{13}C of *Macrocystis* blades incubated under two NO_3^- concentrations (red dot = N-deplete blades, blue triangle = N-replete blades). Each point represents one individual (n = 4 at each temperature treatment (6-27°C)).

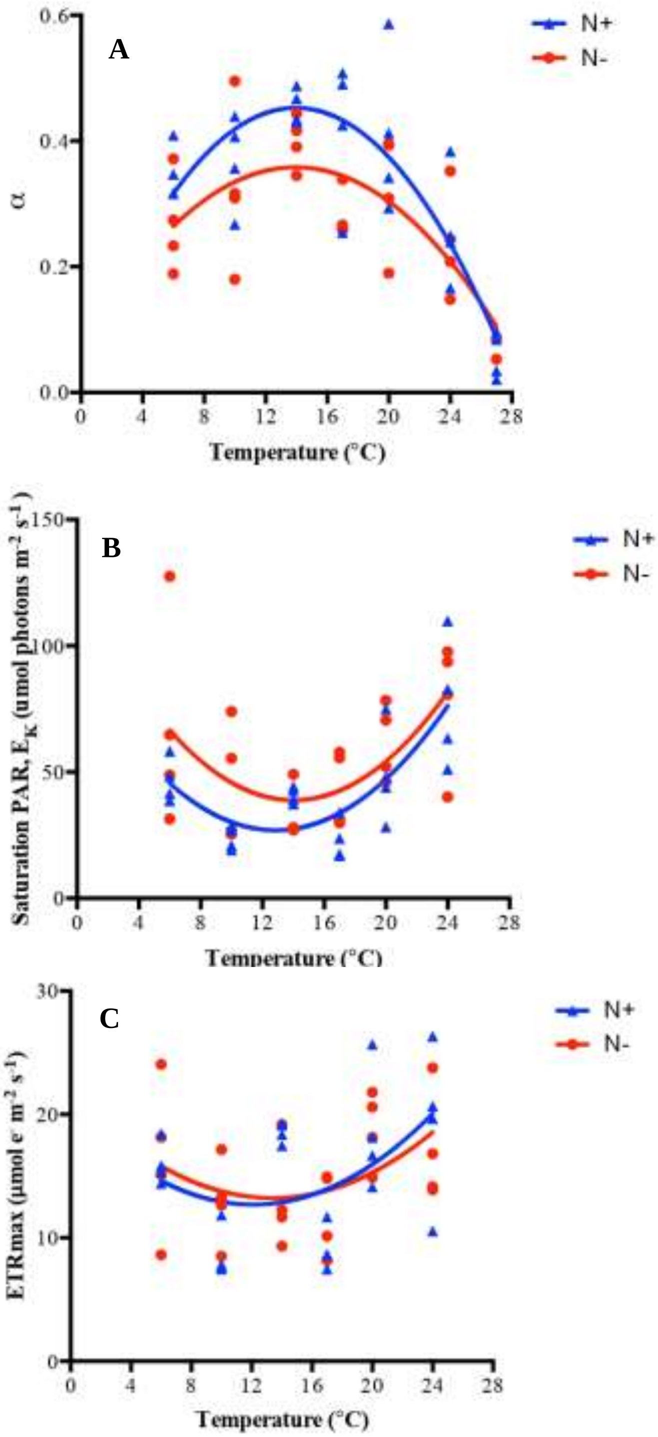

Fig. S3. Photosynthetic parameters (A) α, (B) Ek, (C) ETR_{max} of *Macrocystis* blades incubated under two NO_3^- concentrations (red dot = N-deplete blades, blue triangle = N-replete blades). Each point represents one individual (n = 4 at each temperature treatment (6-27°C)).
